# Volumetric analysis of bilateral spinal canal decompression via hemilaminectomy versus laminoplasty in cervical spondylotic myelopathy

**DOI:** 10.1007/s00701-020-04453-z

**Published:** 2020-06-25

**Authors:** Silvia Hernández-Durán, Noman Zafar, Daniel Behme, Matthias Momber, Veit Rohde, Dorothee Mielke, Ingo Fiss

**Affiliations:** 1 Department of Neurological Surgery, Universitaetsmedizin Goettingen, Robert-Koch-Str. 40, 37075 Goettingen, Germany; 2 Department of Neurological Surgery, Stadt Krankenhaus Korbach gGmbH, Dr.-Hartwig-Str. 19, 34497 Korbach, Germany; 3 Department of Neuroradiology, Universitaetsmedizin Goettingen, Robert-Koch-Str. 40, 37075 Goettingen, Germany; 4 Department of Neurological Surgery, Klinikum Vest GmbH, Dorstener Str. 151, 45657 Recklinghausen, Germany

**Keywords:** Cervical spondylotic myelopathy, Laminoplasty, Laminotomy, Volume gain

## Abstract

**Background:**

Cervical spondylotic myelopathy (CSM) is a degenerative process of the cervical spine requiring surgical decompression to prevent neurological deterioration. While both anterior and posterior approaches yield satisfactory results, posterior decompression is preferred in cases of the multilevel disease. In 2015, we described a muscle-sparing, novel technique of bilateral osteoligamentous decompression via hemilaminectomy (OLD) for CSM. In this study, we investigate whether this technique offers comparable volumetric results to laminoplasty in terms of spinal canal enlargement and whether this technique can yield significant clinical improvement.

**Methods:**

Patients undergoing OLD due to CSM were prospectively enrolled in this study and then matched to and compared with a historic cohort of patients with CSM treated by laminoplasty. An independent sample *t* test was performed to analyze whether the volumetric gain in the two separate groups was statistically significant. Patients in the OLD cohort were clinically evaluated with the mJOA score preoperatively and 3 months postoperatively. To assess clinical improvement, a paired sample *t* test was performed.

**Results:**

A total of 38 patients were included in the analysis: 19 underwent OLD and 19 underwent laminoplasty. Both groups were well matched in terms of sex, age, preoperative spinal canal volume, and involved levels. Both surgical methods yielded statistically significant volumetric gain in the cervical spinal canal, but a trend towards a greater volume gain was seen in the OLD group. In the OLD group, a statistically significant clinical improvement was also demonstrated.

**Conclusions:**

Our study reveals that OLD can yield a comparable extent of decompression to laminoplasty in CSM while also delivering statistically significant clinical improvement.

## Introduction

Cervical spondylotic myelopathy (CSM) is a degenerative process in which the cervical spinal cord (SC) becomes compressed due to osteophytes, disc bulging, yellow ligament hypertrophy, and facet joint arthrosis. Evidence suggests that 20% to 60% of patients will experience neurological deterioration without surgical intervention [8]. While both anterior and posterior approaches yield satisfactory results, posterior decompression is preferred in cases of multilevel disease due to lower complication rates and extensive volume gain [12, 19]. Among the posterior approaches, laminectomy was traditionally used, but this relatively simple procedure has been increasingly controverted because of its association with postoperative kyphotic changes and increased risk for long-term instability [5, 15]. More recently, lateral mass fusion has been added to reduce these delayed postoperative complications, but they involve instrumentation and longer surgical times [10]. As an alternative, laminoplasty (LP) was developed in the 1970s in Japan to preserve the biomechanical function of the CS [11]. Nevertheless, most LP techniques still presuppose bilateral detachment of the nuchal musculature, which can contribute to later instability and axial symptoms [17]. One of the senior authors (D.M.) first described a muscle-sparing, novel technique of bilateral osteoligamentous decompression via hemilaminectomy (OLD) for CSM in 2015 [13]. In this study, we investigate whether this technique offers comparable volumetric results to LP in terms of spinal canal enlargement.

## Materials and methods

### Patient population

In this study, we aimed to determine whether OLD can provide decompression results comparable to those of LP in terms of volume gain. For this purpose, we performed a matched comparison of case series. Patients undergoing OLD due to CSM were prospectively enrolled in this study after approval by our institutional ethics committee (study number 20/2/14). We then matched enrolled patients with a historic cohort of patients with CSM treated by LP at our institution. Patients in the prospective arm were clinically evaluated by means of the modified Japanese Orthopedic Association (mJOA) score preoperatively and 3 months postoperatively to assess myelopathic compromise. In the historic cohort, no standardized clinical scoring was used, which is why the authors refrain from examining this aspect in the LP group.

### Surgical strategy

#### Indications for OLD

At our center, OLD is routinely performed in lieu of any other posterior approaches to treat CSM. OLD is indicated when compression of the cervical spinal cord is due to a dorsal vector, meaning that yellow ligament hypertrophy and/or facet joint arthrosis are the main compressive elements. Furthermore, the lordotic alignment of the cervical spine must be preserved and the diameter of the spinal canal diameter must not be decreased by more than 50%.

#### Surgical technique

**Fig. 1:**
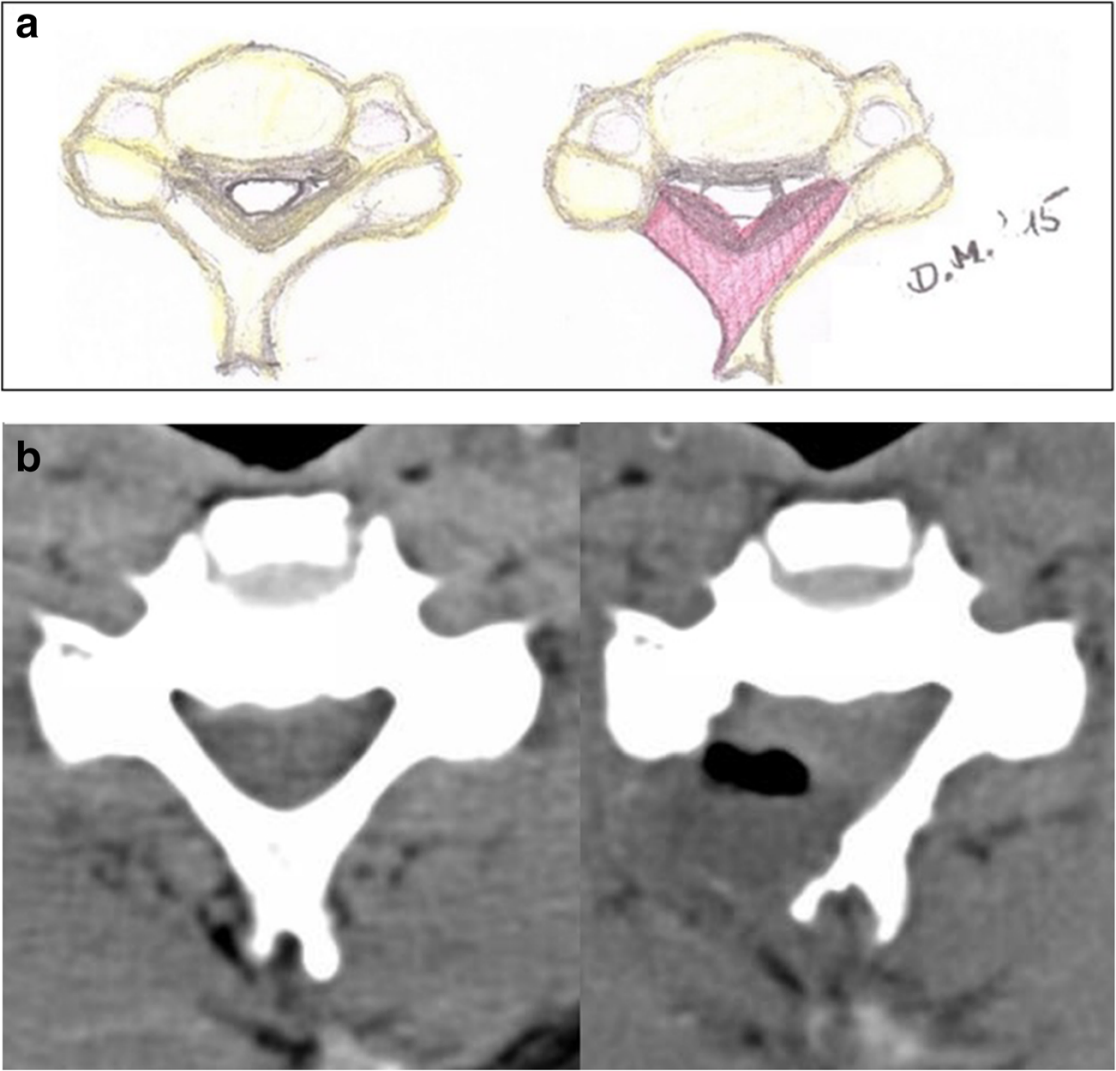
**a***Left* drawing (axial view) of a cervical spinal stenosis caused by a hypertrophied ligamentum flavum and a calcified disc protrusion as well as osteophytes. *Right*: the amount of bone and ligamentum flavum that is being resected is marked in red. This area demonstrates that the contralateral side can be sufficiently decompressed via a unilateral approach. **b** Preoperative (*left*) and postoperative (*right*) axial CT scan of a patient with CSM treated with osteoligamentous decompression (OLD). *Right*: the ipsi- and contralateral side are both sufficiently decompressed via a unilateral, muscle-sparing approach

As previously described by one of the senior authors (D.M.) [13], a novel technique for bilateral spinal canal decompression via hemilaminectomy was employed (Fig. 1 a, b). A hemilaminectomy is performed using a 5-mm high-speed diamond drill. The base of the spinous process is then removed with the diamond drill, beginning at the medial edge of the hemilaminectomy and ending near the contralateral medial part of the facet joint, thereby thinning the inner contralateral hemilamina. Bilateral undercutting of the laminae above and below can then be performed. The yellow ligament is removed with a Kerrison rongeur until the very first segment of the contralateral dorsal nerve root is exposed. Several levels can be treated by using this approach.

**Fig. 2:**
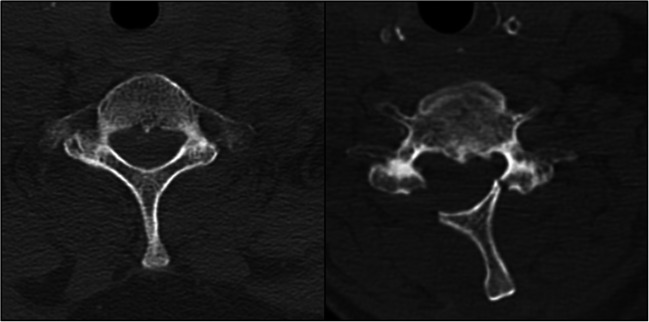
Preoperative (*left*) and postoperative (*right*) axial CT scan of a patient with cervical spondylotic myelopathy treated with laminoplasty (LP). *Right*: according to Hirabayshi’s open-door technique, the laminae are lifted on the hinges to expand the spinal canal. The spinous process was sutured to the paravertebral musculature to ensure a permanently stable position

In the LP group, the open-door technique as described by Hirabayashi was employed [6]. Here, the lamina is bilaterally thinned at the lateral border. On one side, the bone is then completely removed, while on the other side the thinned inner cortex is preserved to function as a hinge. The lamina is then lifted on the hinge to expand the spinal canal. At our institution, the spinous process was then sutured to the paravertebral musculature to ensure the position of the lifted lamina (Fig. 2). LP was also performed on the multilevel disease.

### Volumetric analysis

CT scans were obtained pre- and postoperatively on a Siemens Somatom AS+ 128 slice CT scanner (Siemens Medical Solutions, Forchheim, Germany). Spine imaging was performed according to our institutional standard CT protocol (Care Dose 4D, 120 kV, slice thickness 2 mm, pitch 0.8). Volumetric measurements were performed on a Siemens Syngo Multimodality Workplace, Version VE36A (Siemens Medical Solutions, Forchheim, Germany) using the volume tool. For pre- and postoperative volumetric measurements, 2-mm slices (soft tissue algorithm, convolution kernel B30s) were utilized, whereas the final volume of interest (VOI) was conducted on single-slice VOIs. To restrict volumes to the level of decompression, volumes were measured between the basivertebral foramen of the superior and inferior vertebra relative to the level of decompression. All measurements were performed by a single senior neuroradiologist (D.B.).

### Statistical analysis

One-way ANOVA was performed to determine whether there were any statistically significant differences between the baseline characteristics in the OLD and LP groups. An independent sample *t* test was performed to analyze whether the volumetric gain in the two separate groups was statistically significant. mJOA scores preoperatively and 3 months postoperatively, reported as means with standard deviations, were compared through a paired sample *t* test. Statistical analysis was carried out with IBM® SPSS®, Version 21 (IBM Corp., Armonk, NY, USA). *p* values ≤ 0.05 were considered significant.

## Results

A total of 58 patients had been enrolled in the prospective arm of the OLD study at the time of this analysis. On the other hand, a total of 85 LP patients were identified in our institutional records. Of these, only 19 had preoperative and postoperative imaging available. Thus, we performed case matching based on age, sex, levels involved, and preoperative volume including 19 LP and 19 OLD patients. No statistically significant difference was observed between the two groups, thus showing good matching. The baseline data of both groups is summarized in Table 1.

**Table 1:** Baseline characteristics of patients undergoing osteoligamentous decompression (OLD) and laminoplasty (LP). No statistically significant differences are shown

Characteristic	OLD	LP	*p*
Age in years (range)	58 (45–75)	56 (34–69)	0.42
Sex (F:M)	7:12	9:10	0.52
Mean levels involved (range)	2.32 (1–4)	2.42 (2–4)	0.66
Mean preoperative spinal canal volume (range)	5.51 (1.97–8.53)	6.46 (3.70–9.78)	0.13

The mean age was 58 (range 45–75) in the OLD group, with *n* = 12 (63.2%) males and *n* = 7 (36.8%) females. Operative details of the OLD group are summarized in Tables 2 and 3 In the LP group, mean age was 56 (range 34–69), with *n* = 10 (52.6%) males and *n* = 9 (47.4%) females. Operative details of the LP group are summarized in Tables 4 and 5.

**Table 2:** Level of decompression in the osteoligamentous decompression (OLD) group

Level of decompression in OLD	*N*
C3/4	6
C4/5	14
C5/6	15
C6/7	9
Total levels operated	44

**Table 3:** Extent of decompression in the osteoligamentous decompression (OLD) group

Extent of decompression in OLD	*N*
1 level	2
2 level	11
3 level	4
4 level	2

**Table 4:** Level of decompression in the cervical laminoplasty (LP) group

level of decompression in LP	*N*
C3/4	6
C4/5	12
C5/6	13
C6/7	8
Total levels operated	39

**Table 5:** Extent of decompression in the cervical laminoplasty (LP) group

Extent of decompression in LP	*N*
2 level	12
3 level	6
4 level	1

Volumetric analyses are summarized in Table 6. Both surgical methods yielded statistically significant volumetric gain in the cervical spinal canal. No statistically significant difference in the amount of volume gained through either surgical method was observed, but a trend towards a greater volume gain was seen in the OLD group (Table 7).

**Table 6:** Pre- and postoperative volumetric analyses for laminoplasty (LP) and osteoligamentous decompression (OLD). Either surgical methods yielded statistically significant volumetric gain

	Mean volume preoperative (range)	Mean volume postoperative (range)	Volume gain (in cm^3^)	Volume gain (in %)	*p*
OLD	5.51 (1.97–8.53)	7.58 (3.62–13.03)	2.07 (0.51–4.54)	43 (9–104)	*< 0.001*
LP	6.46 (3.70–9.78)	8.11 (5.41–11.17)	1.65 (0.54–3.77)	29 (9–71)	*< 0.001*

Italic entries contains *p* values < .01 which were significant

**Table 7:** Amount of volume gained through either surgical method. No statistically significant results could be found, but a trend towards a greater volume gain was seen in the OLD group

	OLD	LP	*p*
Volume gain (in cm^3^)	2.07 (0.51–4.54)	1.65 (0.54–3.77)	0.238
Volume gain (in %)	43 (9–104)	29 (9–71)	0.095

Clinically, patients in the OLD group had a mean mJOA score of 12 ± 3 preoperatively. Postoperatively, this value improved to 14 ± 2. This clinical improvement was statistically significant (*p* = .025).

## Discussion

Our study shows that our novel approach yields comparable volumetric results to LP, thus proving our technique to be non-inferior to the current standard of care. In the OLD cohort, we also observed a trend towards a higher volumetric gain, compared with the LP group. We hypothesize that this could be due to the additional undermining of the contralateral hemilamina and spinous process in OLD. Conversely, in our historic cohort, the lifted lamina was sutured to the paravertebral musculature to ensure its position, rendering it susceptible to postoperative shift and reclosure. Studies have shown that spinal canal enlargement after LP is dependent upon LP opening size (LOS) and that this can be greatly improved upon using the appropriate miniplates for lamina fixation [21]. The lack of use of tailored miniplates might have contributed to the inferior volume gain observed in the LP cohort.

The significance of volumetric gain is evinced in a study by Itoh and Tsuji, where a gain in the antero-posterior diameter of the spinal canal of at least 4 mm after LP was necessary to achieve relevant clinical improvement [7]. The advantage of volume gain is not limited to the removal of compression by dorsal components, such as hypertrophied yellow ligaments and arthritic facet joints; anterior compression is also indirectly relieved by providing the SC with space to migrate dorsally [4]. In a study performed by Baba et al., the neurological improvement of patients undergoing LP for CSM was correlated with postoperative dorsal migration of the SC and volumetric gain of the bony spinal canal [1]. Similarly, Sodeyama et al. showed that good recovery rates after LP were significantly correlated to the dorsal shift of the SC in the operated levels [16]. In our study, we were able to demonstrate not only a statistically significant volumetric gain after OLD but also a statistically significant improvement in mJOA scores, thus reflecting the importance of volumetric gain for neurological recovery in CSM and underlining the validity and equipoise of our novel surgical technique.

However, spinal canal volume does not always correlate with clinical symptoms [2] and a key question remains to be answered: can our novel approach hinder the development of kyphosis and cervical instability, the most feared long-term complications of posterior decompressive procedures without stabilization to treat CSM? Several cadaveric biomechanical studies have shown the importance of the extensor musculature to maintain alignment and stability. Nolan et al. found that the semispinalis cervicis and capitis muscles generate considerable force and act as significant dynamic stabilizers of the cervical spine, especially those attached to the occiput and spinous processes of C2 and C7 [14]. Additional studies have underscored the importance of muscle attachment to bony structures to preserve musculoskeletal function, which clinically translates into less postoperative axial pain and decreased risk of kyphosis and instability [9].

Chen et al. compared results of LP with posterior muscle-ligament complex preservation (preservation group) with traditional LP (control group) in patients with CSM [3]. They could show that in the preservation group, postoperative axial symptoms were reduced compared with the control group and concluded that postoperative axial symptoms may arise from posterior muscle-ligament damage. In their preservation group, even an osteotomy at the base of the spinous process was carried out before laminoplasty was performed. Towards the end of the operation, the spinous process was then refixed to the lamina with a screw through the hole of the miniplates and with the help of additional sutures. Compared with our technique of thinning the spinous process and the contralateral inner hemilamina, this procedure seems quite aggressive at first glance with osteotomy of the spinous process but still leads to better clinical results. This can be attributed to the preservation of the musculo-ligamentary complex, which is also preserved according to our technique.

With a focus on the purely biomechanical aspects of the technique, the study of Wu et al. seems worth mentioning [18]. They established an animal model in sheep to assess biomechanical changes after unilateral hemilaminectomy combined with different degrees of facetectomy and concluded that unilateral hemilaminectomy alone as well as unilateral hemilaminectomy with 50% ipsilateral facetectomy does not affect long-term cervical stability. They proposed that only unilateral hemilaminectomy and 100% ipsilateral facetectomy can lead to long-term instability under lateral bending and flexion-extension. Since we pay meticulous attention to the protection of the facet joints, we do not see any evidence of instability induced by our technique from a biomechanical point of view.

When it comes to the consideration of finite element models, we would like to point out the study of Xie et al. [20]. They modified a validated nonlinear finite element model of the intact cervical spine (C2–C7) to study the biomechanical changes of multilevel laminectomy, multilevel hemilaminectomy, and unilateral multilevel interlaminar fenestration with or without unilateral graded facetectomy for the treatment of intradural tumors at the level C3–6. They found that the less invasive approaches of unilateral multilevel interlaminar fenestration and multilevel hemilaminectomy preserved the flexion motion of the cervical spine compared with laminectomy, thus minimizing the risk of postoperative spinal instability and disc degeneration.

By only performing unilateral muscle detachment in addition to the careful preservation of the supra- and interlaminar ligaments, our novel technique should minimize the downsides of the posterior approach by preserving the biomechanical function of the musculoskeletal elements of the cervical spine. These theoretical advantages of OLD remain to be proven through scientific evaluation. An interim analysis of the prospective arm of this study of patients undergoing OLD shows improvement in the Neck Disability Index (NDI) without the development of kyphosis. Nevertheless, long-term follow-up needs to be assessed to provide compelling evidence in favor of this novel surgical technique.

## Conclusion

In sum, our study reveals that OLD can yield a comparable extent of decompression to LP in CSM. Furthermore, patients achieved a statistically significant improvement in their myelopathic symptoms through this novel technique. Further studies are necessary to assess the long-term outcome of patients undergoing OLD for CSM, particularly in terms of axial pain, kyphosis, and instability.
